# Effects of temperature on the cuticular transpiration barrier of two desert plants with water-spender and water-saver strategies

**DOI:** 10.1093/jxb/erz018

**Published:** 2019-01-30

**Authors:** Amauri Bueno, Ahmed Alfarhan, Katja Arand, Markus Burghardt, Ann-Christin Deininger, Rainer Hedrich, Jana Leide, Pascal Seufert, Simona Staiger, Markus Riederer

**Affiliations:** 1 University of Würzburg, Julius von Sachs Institute of Biological Sciences, Chair of Botany II – Ecophysiology and Vegetation Ecology, Würzburg, Germany; 2 Department of Botany and Microbiology, College of Science, King Saud University, Riyadh, Saudi Arabia; 3 University of Würzburg, Julius von Sachs Institute of Biological Sciences, Chair of Botany I – Molecular Plant Physiology and Biophysics, Würzburg, Germany

**Keywords:** *Citrullus colocynthis*, cuticular transpiration, cuticular waxes, drought, *Phoenix dactylifera*, thermal stress

## Abstract

Water-saver and water-spender strategies are successful adaptations allowing plants to cope with the limitations of hot desert habitats. We investigated whether the efficacy of the cuticular transpiration barrier and its susceptibility to high temperatures are ecophysiological traits differentially developed in the water-spender *Citrullus colocynthis* and the water-saver *Phoenix dactylifera*. Minimum leaf conductance (*g*_min_) at 25 °C was six times lower in *P. dactylifera* (1.1×10^–5^ m s^–1^) than in *C. colocynthis* (6.9×10^–5^ m s^–1^). Additionally, *g*_min_ in the range 25–50 °C did not change in *P. dactylifera* but increased by a factor of 3.2 in *C. colocynthis*. Arrhenius formalism applied to the *C. colocynthis g*_min_ led to a biphasic graph with a steep increase at temperatures ≥35 °C, whereas for *P. dactylifera* the graph was linear over all temperatures. Leaf cuticular wax coverage amounted to 4.2±0.4 µg cm^–2^ for *C. colocynthis* and 29.4±4.2 µg cm^–2^ for *P. dactylifera*. In both species, waxes were mainly composed of very-long-chain aliphatics. Midpoints of the wax melting ranges of *P. dactylifera* and *C. colocynthis* were 80 °C and 73 °C, respectively. We conclude that in *P. dactylifera* a particular wax and cutin chemistry prevents the rise of *g*_min_ at elevated temperatures.

## Teaser

The efficacy of the cuticular transpiration barrier and its resistance to elevated temperatures are significantly higher in a typical water-saver than in a water-spender plant growing in hot desert.

## Introduction

Desert plants have evolved a wide range of adaptations that allow them to cope with water shortage and intense solar radiation. There are numerous strategies for dealing with drought stress, and plants can be classified along an ‘adaptation continuum’ (of resistance) from drought tolerance to drought avoidance (Smith *et al.*, 1997). Baquedano and Castillo (2006) showed that drought-avoiding plants utilize two contrasting strategies in response to the scarcity of water. (i) water-saver plants close their stomata early after the onset of drought and thereby prevent damage before the leaf water potential changes substantially; (ii) water-spender plants, by contrast, keep their stomata open for prolonged periods of drought and thus decrease the leaf water potential, allowing them to extract water from the soil to compensate for the high water loss by stomatal transpiration (Guehl and Aussenac, 1987; Lo Gullo and Salleo, 1988).

The stomata and cuticle are the primary components responsible for maintaining a favourable plant water status. At maximum stomatal closure, the water permeability of the cuticle determines the minimum and inevitable water loss and, therefore, is one of the principal features ensuring the successful establishment and survival of plants. The cuticle, a cutin matrix with cuticular waxes embedded within it (intracuticular waxes) and deposited on its surface (epicuticular waxes), covers all primary aerial parts of terrestrial higher plants. The cutin matrix primarily consists of cross-linked C_16_ and C_18_ hydroxy and hydroxy epoxy alkanoic acids, whereas the characteristic cuticular waxes comprise very-long-chain (VLC) aliphatic and cyclic components (Jetter *et al.*, 2006). Published values of leaf cuticular permeability reviewed by Schuster *et al.* (2017) vary among plant species from 2.6×10^–7^ m s^–1^ (*Zamioculcas zamiifolia*) to 2.5×10^–4^ m s^–1^ (*Helianthus anomalus*). For comparison, representative values of stomatal conductances of mesophytes lie between 4×10^–3^ m s^–1^ and 20×10^–3^ m s^–1^, while for xerophytes and trees they range from 1×10^–3^ m s^–1^ to 4×10^–3^ m s^–1^ (Nobel, 2009) and, consequently, are one to four orders of magnitude higher than cuticular water permeabilities. The extraction of the cuticular waxes increases the cuticular water permeability by up to several orders of magnitude (Schönherr, 1976; Schönherr and Lendzian, 1981), showing that the waxes make up the main barrier to transpiration. However, so far, there is no evidence that the cuticle thickness or the amount of cuticular waxes per unit area reduces the cuticular water permeability (Schreiber and Riederer, 1996; Riederer and Schreiber, 2001).

In hot deserts, drought often co-occurs with high temperatures. Over the course of the day, relative air humidity can reach low values as the temperature increases, leading to a concomitant rise of the water vapour pressure deficit. Therefore, the efficient control of water loss at high temperatures is of fundamental importance for ensuring the survival, viability, and reproductive fitness of desert plants.

Consequently, the effect of temperature on the cuticular water permeability is a decisive parameter determining the physiological and ecological role the cuticle plays, especially in conditions of high evaporative demand and elevated temperature. The cuticular permeability of all non-desert plants studied so far increased slightly at temperatures from 15 °C to 35 °C, while temperatures above 35 °C caused a drastic increase in permeability (Schreiber, 2001; Riederer, 2006). In contrast to the non-desert plants studied so far, the cuticular water permeability of the desert plant *Rhazya stricta* increased only slightly between 15 °C and 50 °C, without an abrupt change in slope at higher temperatures (Schuster *et al.*, 2016).

In this study, we investigated the leaf cuticular transpiration barrier and its susceptibility to thermal effects as potential adaptations to aridity and high evaporative demand under elevated temperature in two plant species from the Arabian desert. *Citrullus colocynthis* (L.) Schrad. (Cucurbitaceae) and *Phoenix dactylifera* L. (Arecaceae) were chosen as models as both species often grow near each other under identical climatic conditions characterized by frequent periods of intense irradiation, air temperatures of up to 50 °C, and high evaporative demand. *C. colocynthis* is a broad-leaved summergreen vine, which spreads its trailing stems along the soil surface. Its leaves stand only a few centimetres above the ground (Lange, 1959). Stem growth begins with buds on the taproots of established plants, and after fruit production, the aboveground parts die off (Cunningham *et al.*, 2011). *P. dactylifera*, by contrast, is a long-lived evergreen woody plant that grows up to 30 m height. The straight leaves are obliquely vertically oriented and form a crown at the end of the trunk (Barrow, 1998).

Desert soil surfaces may heat up to extreme temperatures owing to the intense solar radiation. Consequently, trailing plants and other plants with leaves close to the soil surface can be exposed to intense heat stress. A pioneer in desert plant ecology, Lange (1959), pointed out the contrasting ecophysiological strategies of the two species studied here regarding water relation and thermal tolerance. He characterized *C. colocynthis* as a typical perennial water-spender plant, which is heat avoidant and possesses a deep root system and short-lived leaves (Si *et al.*, 2009). Even under intense solar radiation, an actively transpiring leaf of *C. colocynthis* maintained its temperature 10–15 °C below that of the surrounding air and far from the limit of heat tolerance of this species, thereby avoiding death by overheating (Lange, 1959; Althawadi and Grace, 1986). The temperature of a cut leaf separated from the water supply, after a short time, exceeded the air temperature and even reached temperatures higher than 60 °C (Lange, 1959). The author associated this strong cooling effect in *C. colocynthis* with high transpiration rates and consequently high water consumption. In contrast to *C. colocynthis*, *P. dactylifera* is a typical woody water-saver plant (Lange, 1959) with evergreen leaflets that are able to thrive in hot and dry conditions as long as there is sufficient moisture around its roots (Barrow, 1998). Leaflets of *P. dactylifera* exposed to high solar irradiation reached temperatures above that of the air temperature. Despite this leaflet overheating, their temperature did not exceed the limit of leaflet heat tolerance (59 °C), ensuring their survival (Lange, 1959). Surprisingly, it has not yet been investigated whether the efficacy of the cuticular transpiration barrier against water loss and its susceptibility to thermal damage are also traits particular to the water-saver and water-spender strategies.

We hypothesize that water-saver, as compared with water-spender, plants in hot arid environments should have (i) a cuticular transpiration barrier with a lower water permeability and consequently a higher efficacy in reducing transpiration when stomata are closed, and (ii) a cuticular transpiration barrier that is less susceptible to adverse thermal effects. To test these hypotheses, we re-investigated two of the plant species Lange (1959) had used in his pioneering work. The minimum leaf water conductance (*g*_min_), as a proxy for cuticular permeability, of *C. colocynthis* and *P. dactylifera* was measured within the range of ecologically relevant temperatures (Almazroui *et al.*, 2014). In the past, it has been shown that *g*_min_ determined by the method used in the present study is a good proxy for cuticular permeability with only very small or no contributions of residual transpiration through potentially leaky stomata (Burghardt and Riederer, 2003; Burghardt *et al.*, 2008; Schuster *et al.*, 2017).

Additionally, we documented the fine structure of the leaf surface using scanning electron microscopy. The qualitative and quantitative compositions of the cuticular waxes and the cutin matrix of both species were evaluated chromatographically. The melting behaviour of the cuticular waxes of both species was investigated by Fourier transform infrared spectroscopy (FTIR). Combining the data on the functional and chemical properties of the cuticle of both plant species, we propose a novel hypothesis relating the chemical composition and phase behaviour of the cuticular waxes of both species to the thermal resistance of their cuticular transpiration barrier.

## Materials and methods

### Plant material and growing conditions

Seeds of *Citrullus colocynthis* (L.) Schrad. were harvested from a natural population growing near Riyadh, Saudi Arabia. *Phoenix dactylifera* L. plants approximately 5 years old and 2 m high were purchased from a commercial supplier (‘Der Palmenmann’, Bottrop, Germany). Plants were cultivated in a greenhouse at 24 °C/18 °C and 50±5% relative humidity day/night under a 14 h light regime at 250 µmol m^–2^ s^–1^ white light (Philips Master Agro, 400 W). Healthy, fully expanded current-year leaves (laminae) of *C. colocynthis* and leaflets (pinnae) from 1-year-old leaves of *P. dactylifera* were harvested and used for the experiments.

### Morphological leaf traits

Before measurement, leaves were rehydrated in a glass desiccator overnight by immersing their petioles in water. The saturation leaf weights were determined using an analytical balance (MC-1 AC210S, Sartorius; precision 0.1 mg). Dry weight was measured after oven-drying the leaves at 90 °C for 24 h. The actual fresh weights during leaf drying experiments were used to calculate the relative water deficit (RWD) according to:

$$RWD=1-\frac{actual\;fresh\;weight-dry\;weight}{saturated\;weight-dry\;weight}$$ (1)

For determination of leaf area, leaves were scanned at high resolution (600 dpi) using a flatbed scanner. The projected leaf area was measured from the scanned leaf image using Adobe Photoshop image analysis software. Leaf mass per area (LMA) was calculated by dividing the dry weight by the leaf area. The leaf water content was calculated by subtracting the dry weight from the saturation leaf weight and subsequently dividing the value obtained by the saturation weight.

### Scanning electron microscopy

The structure of the leaf cuticular surface was characterized using scanning electron microscopy. Small air-dried pieces (2–3 mm) of fully developed leaves of *C. colocynthis* and leaflets of *P. dactylifera* were mounted on aluminium stubs using double-sided adhesive tape and sputter-coated with 10–15 nm gold-palladium (150 s, 25 mA, partial argon pressure 0.05 mbar, SCD005 sputter coater, Bal-Tec). The samples were investigated with a field-emission scanning electron microscope (JEOL JSM-7500F) using a 5 kV acceleration voltage and a 10 mm working distance. Pictures were taken from both adaxial and abaxial leaf surfaces.

### Minimum leaf water conductance

Leaf minimum water conductance (*g*_min_) represents the lowest water loss a leaf reaches when its stomata are maximally closed due to desiccation stress (Körner, 1995). *g*_min_ was determined gravimetrically from the consecutive weight loss of desiccating leaves in an incubator in the dark and with controlled very low air humidity (Burghardt and Riederer, 2003). High-melting-point (68 °C) paraffin wax (Fluka) was used to seal the wounds of cut petioles of water-saturated leaves and leaflets. Subsequently, the sealed leaves were placed in an incubator (IPP 110, Memmert) with controlled temperature. For evaluating the effect of temperature, *g*_min_ was measured at 25 °C, 30 °C, 35 °C, 40 °C, 45 °C, and 50 °C. The air temperature and humidity in the incubator were monitored with a digital thermo-hygrometer (Testoterm 6010, Testo). Silica gel (Applichem) was used to control the air humidity in the incubator. The actual weight of desiccating leaves was determined as a function of desiccation time using a high-precision balance (MC-1 AC210S, Sartorius). The transpiration rate (*J*) was calculated from the change in fresh weight (ΔFW) with time (t) divided by the total projected leaf area (sum of the adaxial and abaxial projected areas, A):

$$J=\frac{\Delta FW}{\Delta t\times A}$$ (2)

Cuticular water conductance was calculated from *J* divided by the driving force for water loss from the outer epidermal cell wall to the surrounding air. The driving force for vapour-based conductance is defined by the difference between the saturation concentrations of water vapour at the temperatures of the leaf (*C*_wv sat leaf_) and the surrounding atmosphere (*C*_wv sat air_) multiplied by the water activities in the epidermal apoplast (α_apo_) and the air (α_lair_):

$$g=\frac{J}{\alpha_{apo}\times C_{wv\;sat\;leaf}-\alpha_{air}\times C_{wv\;sat\;air}}$$ (3)

The water activity of the air (α_air_) over silica gel is nearly zero (Slavík, 1974) and the water activity in the apoplast adjacent to the inner side of the cuticle (α_apo_) can be assumed to be close to 1. Thus, the active driving force for cuticular transpiration in the setup used here is the saturation concentration of water vapour at the actual leaf temperature (*C*_wv sat leaf_; Nobel, 2009). Leaf temperatures were measured at 30 min intervals using an infrared (IR) laser thermometer (Harbor Freight Tools, one-point measurements), and the corresponding water vapour saturation concentrations at leaf temperature were derived from tabulated values (Nobel, 2009).

The leaf conductance was plotted against the corresponding RWD. The conductance was high at low RWDs and declined with progressive leaf desiccation until reaching a plateau where the conductance did not decline further with RWD. This constant low conductance corresponds to *g*_min_ and characterizes the leaf water loss at maximum stomatal closure caused by cuticular transpiration (Burghardt and Riederer, 2006; Schuster *et al.*, 2016, 2017).

### Chemical composition of cuticular waxes

Cuticular waxes of both species were extracted from leaves of the same age as those used in the determination of morphological leaf traits and cuticular transpiration. The extraction conditions appropriate for each species had been established by prior tests. *C. colocynthis* leaves were dipped (avoiding the wounds of cut petioles) twice into 10 ml chloroform (≥99.8%, Roth) for 1 min at room temperature. Leaflets of *P. dactylifera* were immersed (avoiding wounds) twice in 25 ml chloroform (≥99.8%, Roth) and kept for 5 min in an ultrasonic bath at room temperature. *N*-tetracosane (C_24_; ≥99.5%, Sigma-Aldrich) was added as an internal standard, and the solutions were reduced to dryness under a gentle flow of nitrogen.

Dry wax samples were derivatized for gas chromatography with *N*,*O*-bis(trimethylsilyl)trifluoroacetamide (BSTFA, Marchery-Nagel) in anhydrous pyridine (≥99.5%, Roth) for 30 min at 70 °C. Quantification of cuticular wax components was performed with a gas chromatograph equipped with a flame ionization detector and an on-column injector (7890A, Agilent Technologies). Separation of compounds was carried out on a fused-silica capillary column (DB1-ms, 30 m length × 0.32 mm internal diameter, 0.1 µm film, Agilent Technologies) with hydrogen as a carrier gas. The temperature programme consisted of injection at 50 °C for 2 min, then raised by 40 °C min^–1^ to 200 °C, held at 200 °C for 2 min, and then raised by 3 °C min^–1^ to 320 °C, and held at 320 °C for 30 min. Single compounds were quantified against the internal standard. Qualitative analysis was carried out using a gas chromatograph equipped with a mass spectrometric detector (5975 iMSD, Agilent Technologies) following the same gas chromatographic conditions, except that helium was the carrier gas. Identification of all the compound classes for both species was achieved by comparing a query mass spectrum with reference mass spectra in a library (NIST05) via spectrum matching.

Weighted median carbon chain-lengths (MCL) for the different wax samples were calculated to characterize the chain-length distributions of VLC aliphatic compounds. Molar coverages for each component were calculated from the gas chromatographic data and summed up according to carbon chain-lengths. For each chain-length *N*_*i*_ the molar fraction *w*_*i*_ was obtained and used as a weight for further calculating the MCL. For *n* distinct ordered chain-lengths *N*_*1*_, *N*_*2*_, … *N*_*n*_ with weights *w*_*1*_, *w*_*2*_, … *w*_*n*_, the MCL is the chain-length *N*_*k*_ satisfying

$$\sum_{i=1}^{k-1}w_{i}\leq \frac{1}{2}\;and\;\sum_{i=k+1}^{n}w_{i}\leq \frac{1}{2}$$ (4)

### Chemical composition of the cutin matrix

For cutin depolymerization, delipidated leaves of *C. colocynthis* and leaflets of *P. dactylifera* were transesterified with boron trifluoride in methanol (Fluka) at 70 °C overnight to release methyl esters of cutin acid monomers. Sodium chloride-saturated aqueous solution (AppliChem), chloroform, and *n*-dotriacontane (C_32_; Sigma-Aldrich) as an internal standard were added to all reaction mixtures. From this two-phase system, the de-esterified cutin components were extracted three times with chloroform. The combined organic phases were dried over anhydrous sodium sulfate (AppliChem). All extracts were filtered, and the organic solvent was evaporated under a continuous flow of nitrogen.

Derivatization and subsequent gas chromatographic analysis with mass spectrometric detection were performed according to Leide *et al.* (2011). Separation of cutin mixtures was carried out at 50 kPa for 60 min, increased by 10 kPa min^–1^ to 150 kPa, and at 150 kPa for 30 min using a temperature programme of 50 °C for 1 min, raised by 10 °C min^–1^ to 150 °C, held at 150 °C for 2 min, and then raised by 3 °C min^–1^ to 320 °C and held at 320 °C for 30 min. The quantitative composition of the mixtures was studied using capillary gas chromatography and flame ionization detection under the same gas chromatographic conditions.

### Melting of cuticular waxes

The melting of the leaf cuticular waxes of *C. colocynthis* and *P. dactylifera* was analysed by FTIR according to the method developed by Merk *et al.* (1997). A Bruker Tensor 27 FTIR spectrometer with a BIO-ATR II® unit (Bruker, Ettlingen, Germany) was used for recording the spectra in horizontal attenuated total reflection mode (h-ATR). The BIO-ATR II® unit consists of a silicon crystal in a stainless steel holder. The wax solution in chloroform (dry weight of a single sample ~200 µg) was deposited on the crystal at 35 °C, and the solvent was quantitatively evaporated. Subsequently, the crystal was cooled to 24 °C for IR measurement. IR spectra were recorded in the wavenumber range from 4000 to 670 cm^–1^ at temperatures from 24 °C to 94 °C. The temperature of the holder was controlled by connecting it to a thermostat (Thermo Scientific Haake DC30-K20, Karlsruhe, Germany). The BIO-ATR II® unit was purged continuously with dry CO_2_-free air (K-MT-LAB 3, Parker Hannifin, Kaarst, Germany). The initial temperature was set to 24 °C and was increased to 48 °C in intervals of 4 °C . From 48 °C to 94 °C, IR spectra were recorded at temperature intervals of 1 °C . The resolution was set to 2 cm^–1^ with an acquisition time of 120 scans. OPUS 7 software (Bruker, Ettlingen, Germany) was used to control the spectrometer and the thermostat, as well as to analyse the spectra. The maxima of the CH_2_ asymmetric stretching bands were determined in the range from 2195 to 2922 cm^–1^ and used for determination of the wax melting ranges (Merk *et al.*, 1997).

### Statistical analysis

Statistical analyses were performed using SPSS Statistics software version 23.0 (IBM Corporation). Normal distribution of data was tested using the Shapiro–Wilk test. Subsequently, a *t*-test was used to compare the minimum conductances of both plant species at 25 °C. Spearman rank correlation was performed to investigate the impact of temperature increase on *g*_min_. Quantitative differences between the cuticular wax coverage of the species were investigated using *t*-tests or Mann–Whitney *U*-tests depending on the dataset distribution.

## Results

### Morphological leaf traits

The morphological traits of *C. colocynthis* leaves and *P. dactylifera* leaflets are shown in Table 1.

**Table 1.** Morphological traits of *Citrullus colocynthis* leaves and *Phoenix dactylifera* leaflets

Trait	*Citrullus colocynthis*	*Phoenix dactylifera*
Total projected leaf area ^*a*^ (×10^–2^ m^2^)	0.45±0.13	0.74±0.25
Saturation leaf weight (g)	0.82±0.34	1.27±0.42
Dry weight (g)	0.14±0.06	0.59±0.21
Leaf water content (g g^–1^)	0.83±0.02	0.54±0.03
Leaf mass per area (g m^–2^)	60.11±15.95	161.83±22.41

^*a*^ Sum of projected adaxial and abaxial leaf surfaces.

Each value represents the mean ±SD (*n*≥61).

### Leaf surface properties

**Fig. 1.**
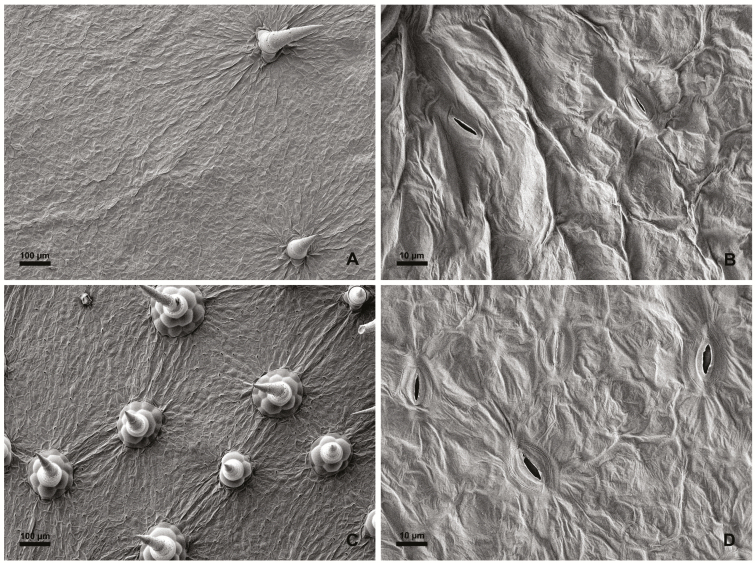
Scanning electron micrographs of the adaxial (A, B) and abaxial (C, D) surface of *Citrullus colocynthis* leaves.

**Fig. 2.**
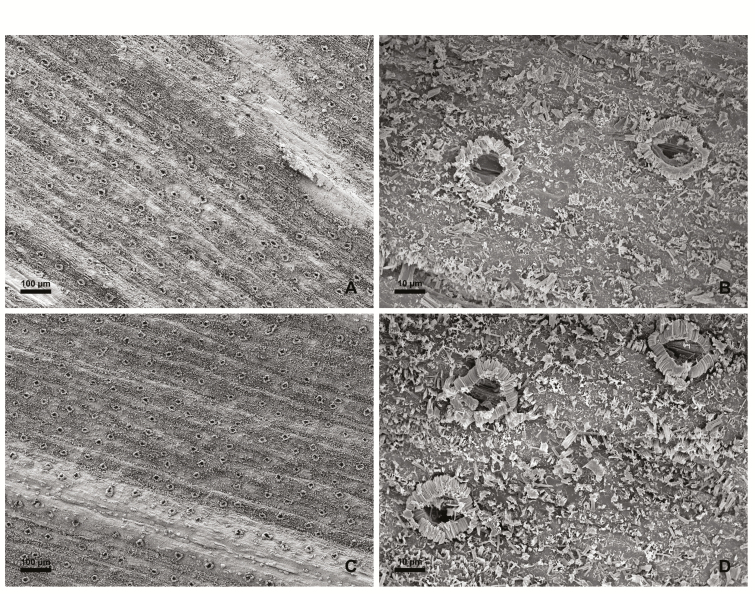
Scanning electron micrographs of the adaxial (A, B) and abaxial (C, D) surface of *Phoenix dactylifera* leaflets.

Leaves of *C. colocynthis* and leaflets of *P. dactylifera* were analysed by scanning electron microscopy to examine the morphology of the leaf surface. The presence of stomata, trichomes, and epicuticular wax structures were the primary features investigated. Both plant species had stomata on both the adaxial and abaxial leaf surfaces (Figs 1 and 2). Stomata of *C. colocynthis* were distributed without any distinct pattern over the whole leaf surface. The cuticular surface was smooth without any explicit crystalline epicuticular deposits (Fig. 1B, D) and the density of trichomes varied among the adaxial (Fig. 1A) and abaxial (Fig. 1C) surfaces. In contrast, the stomata of *P. dactylifera* were arranged in rows along both the adaxial (Fig. 2A) and abaxial (Fig. 2C) leaflet surfaces. Chimney-like cuticular wax surrounded the stomata (Fig. 2B, D), leading to a locally increased boundary layer resistance affecting only stomatal transpiration. Trichomes could not be detected.

### Leaf drying curves and minimum leaf water conductance

The actual leaf temperature at a given temperature of the surrounding air was measured to calculate the driving force for cuticular transpiration. Leaf temperatures of both *C. colocynthis* and *P. dactylifera* were slightly lower than the corresponding air temperatures at 25 °C and 30 °C. With rising air temperatures, the leaf–air temperature differences (Δ*T*_leaf–air_) of *C. colocynthis* increased continuously until reaching a value of approximately –4 °C at an air temperature of 50 °C. The ΔT_leaf–air_ of *P. dactylifera* remained at approximately –1 °C at all temperatures above 30 °C (see Supplementary Table S1 at *JXB* online). Minimum cuticular transpiration rates (*J*_min_) increased with air temperature. *J*_min_ rose from 1.3±0.4×10^–3^ g m^–2^ s^–1^ at 25 °C to 13.2±2.8×10^–3^ g m^–2^ s^–1^ at 50 °C in *C. colocynthis*, and from 0.2±0.0×10^–3^ g m^–2^ s^–1^ to 1.0±0.2×10^–3^ g m^–2^ s^–1^ in *P. dactylifera* (Supplementary Table S1).

**Fig. 3.**
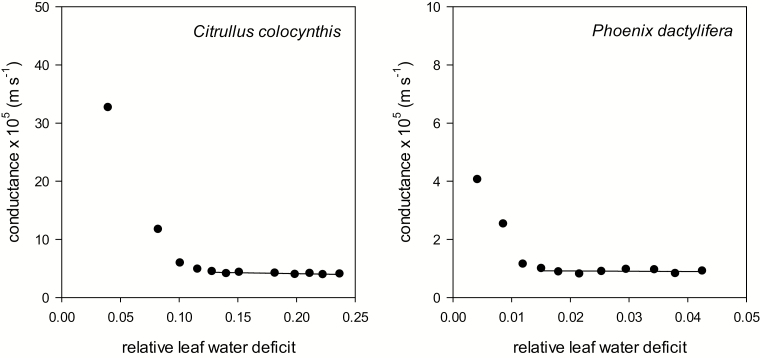
Leaf conductance to water vapour as a function of the relative water deficit (RWD). The drying curves shown are representative for a leaf of *Citrullus colocynthis* (A) and a leaflet of *Phoenix dactylifera* (B) at 25 °C. The RWD at maximum stomatal closure is indicated by the transition between the declining phase and the plateau phase of leaf conductance. After maximum stomatal closure, leaf conductance remains constant at a minimum, defined as the minimum leaf conductance (*g*_min_).

The kinetics of water loss from detached leaves of *C. colocynthis* and leaflets of *P. dactylifera* exposed to dry air were measured over time. Conductances were calculated from the water loss rates for each pair of consecutive data points along the leaf drying curve. For both plant species, the conductance was high in the initial phase of leaf drying. During leaf dehydration, the conductance declined to reach a plateau yielding the minimum conductance (*g*_min_) at maximum stomatal closure (Fig. 3). Under such conditions, water loss became linear over time (Supplementary Fig. S1). Leaf drying curves at 50 °C showed essentially the same pattern as those at 25 °C for *P. dactylifera*, but not for *C. colocynthis*. The conductance of *C. colocynthis* at 50 °C, after the initial phase, progressively decreased until a late stomatal closure, when the RWD reached a value of ~0.6 (Table 2).

**Table 2.** Minimum conductance (*g*_min_) and relative water deficit at maximum stomatal closure (RWD_SC_) of *Citrullus colocynthis* leaves and *Phoenix dactylifera* leaflets obtained from leaf drying curves as functions of air temperature (*T*_air_)

*T*_air_ (°C)	*g*_min_×10^5^ (m s^–1^)		RWD_SC_	
	*C. colocynthis*	*P. dactylifera*	*C. colocynthis*	*P. dactylifera*
25	6.94±2.00	1.11±0.24	0.14±0.05	0.014±0.004
30	7.83±1.23	0.92±0.16	0.15±0.06	0.010±0.006
35	7.74±1.46	1.00±0.12	0.16±0.03	0.014±0.001
40	8.79±2.41	1.19±0.47	0.20±0.08	0.011±0.006
45	13.70±5.33	1.37±0.32	0.30±0.08	0.015±0.003
50	21.98±5.19	1.30±0.26	0.60±0.04	0.016±0.005

Each value represents the mean ±SD (*n*≥8).

The transition point representing the RWD at the onset of maximum stomatal closure (RWD_SC_) was calculated from the intersection of the steep declining phase and the plateau phase of the leaf drying curves. The two plant species showed different responses in terms of stomatal closure. In the temperature range from 25 °C to 40 °C, *C. colocynthis* maximally closed the stomata at RWDs between 0.1 and 0.2. At higher temperatures, maximum stomatal closure shifted to higher RWDs of up to 0.6 at an air temperature of 50 °C. In comparison, *P. dactylifera* maximally closed the stomata at RWD values 10 times lower than in *C. colocynthis*, regardless of the air temperature (Table 2).

**Fig. 4.**
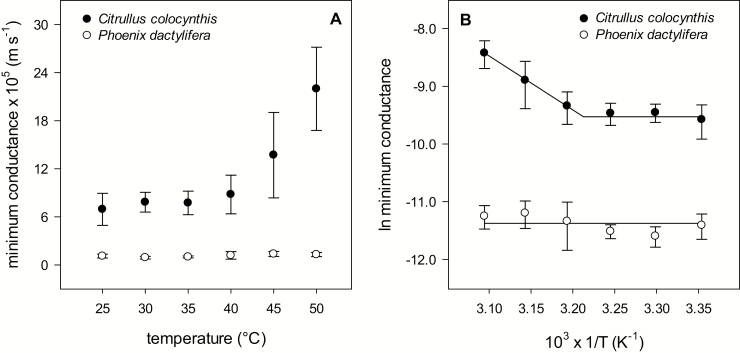
Minimum conductance (*g*_min_; A) and Arrhenius plots of the *g*_min_ (B) of *Citrullus colocynthis* leaves and *Phoenix dactylifera* leaflets obtained from drying curves as a function of air temperature (*n*≥8). Actual leaf temperatures deviated from the corresponding air temperature by a maximum of 4 °C. For calculating *g*_min_ the driving force derived from the actual leaf temperature was used. The Arrhenius formalism applied to the *g*_min_ of *C. colocynthis* led to a biphasic graph with no statistically significant (*P*=0.383) change of conductance from 25 °C to 35 °C and a sharp significant increase (*P*=0.017) at higher temperatures (*R*^2^=0.99, ln *g*_min_=20.3–9.3×10^3^ 1/T). In *P. dactylifera*, there is no statistically significant change (*P*=0.087) of the conductance over the whole range of temperatures, and no transition.

The *g*_min_ of *C. colocynthis* at 25 °C, 6.9±2.0×10^–5^ m s^–1^, was more than six times higher [*t* (14.6)=–10.8, *P*<0.001] compared with the *g*_min_ of *P. dactylifera* at the same temperature (1.1±0.2×10^–5^ m s^–1^). The *g*_min_ of *C. colocynthis* increased with temperature by a factor of 3.2 between 25 °C and 50 °C, from 6.9±2.0×10^–5^ m s^–1^ to 22.0±5.2×10^–5^ m s^–1^. In comparison, the *g*_min_ of *P. dactylifera* remained constant over the whole temperature range. The *C. colocynthis g*_min_ did not change significantly between 25 °C and 35 °C but then steeply increased at temperatures above 35 °C (Fig. 4A).

### Chemical composition of cuticular waxes

**Fig. 5.**
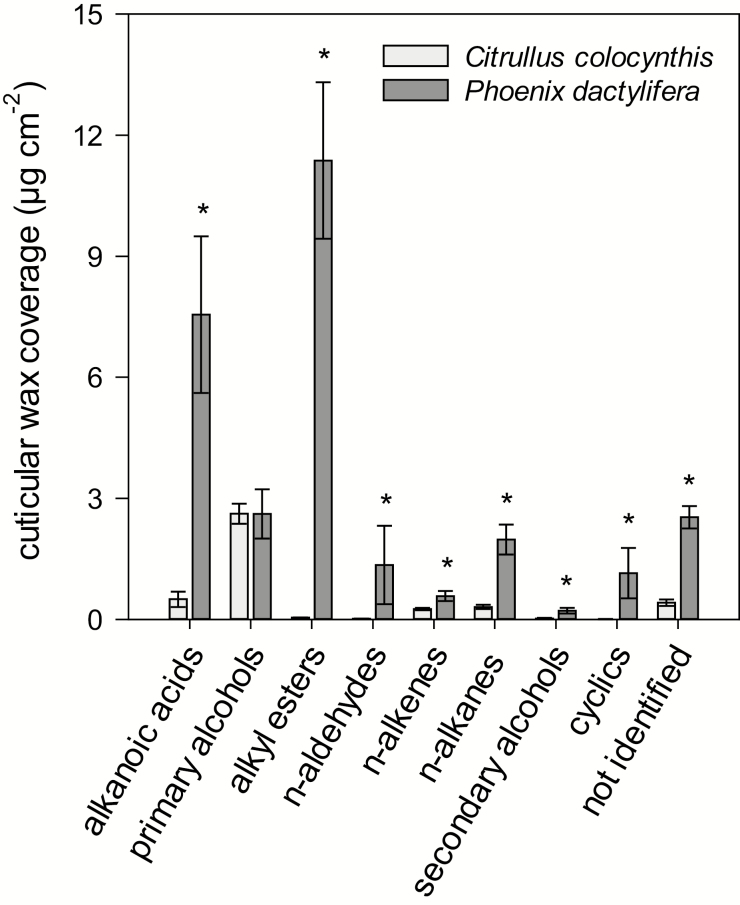
Cuticular wax coverage of *Citrullus colocynthis* leaves and *Phoenix dactylifera* leaflets arranged by compound class. Each value represents the mean ±SD (*n*≥3). Asterisks indicate significant differences in cuticular wax coverage between the species (*P*<0.05).

**Fig. 6.**
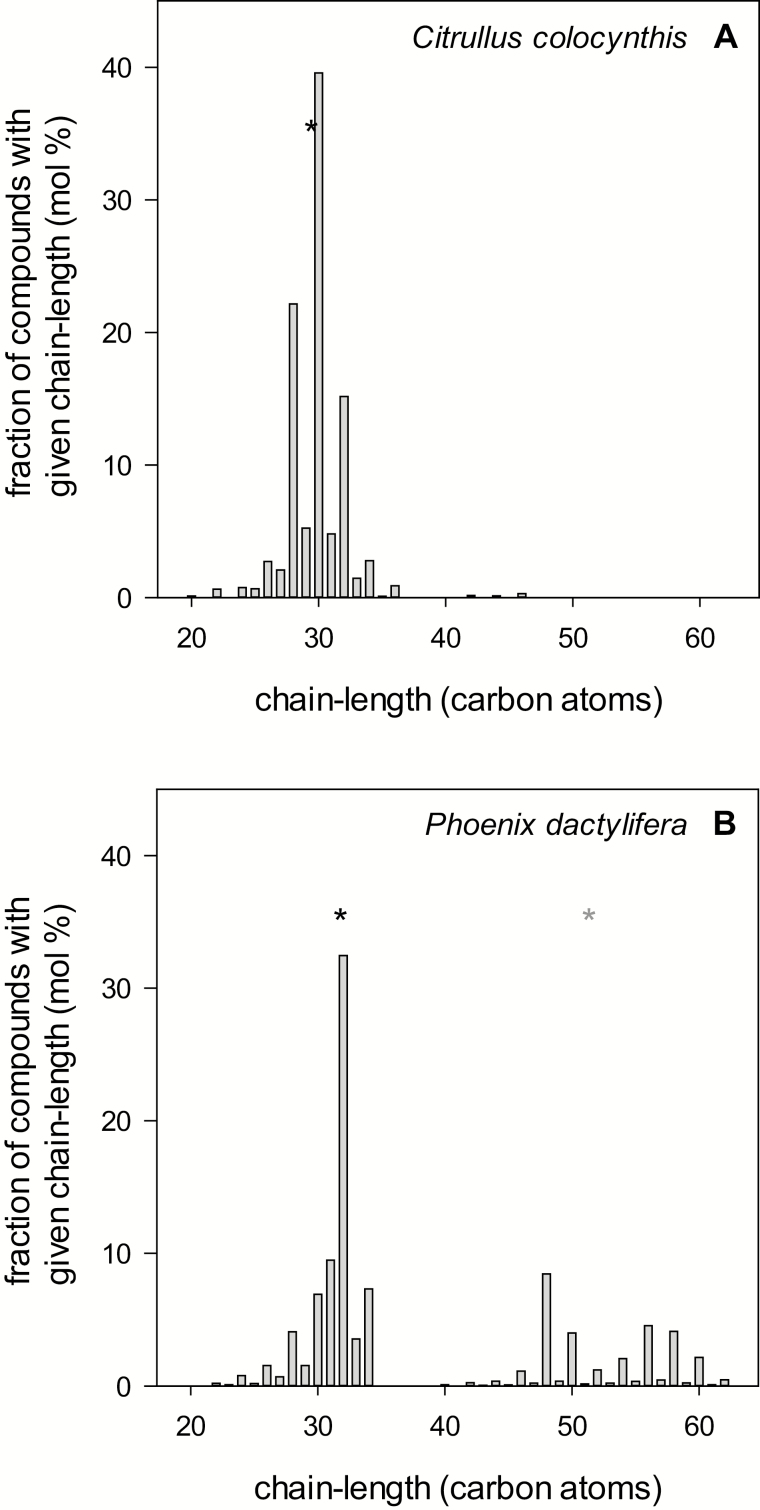
Chain-length distributions of the very-long-chain (VLC) aliphatic compounds of cuticular waxes from leaves of *Citrullus colocynthis* (A) and leaflets of *Phoenix dactylifera* (B). Bars represent the mole-based contribution of a single chain-length to the total VLC fraction. Asterisks denote the weighted median carbon chain-lengths (MCL; the 50% weighted percentile of the chain-lengths) of the VLC aliphatic fraction with chain-lengths <40 (black asterisk) and ≥40 (grey asterisk) carbon atoms.

The cuticular waxes of *C. colocynthis* leaves and *P. dactylifera* leaflets were analysed to investigate differences between the plant species and detect potential relationships between cuticular transpiration and the cuticular wax quantity and composition. The leaf cuticular wax coverage was 4.2±0.4 µg cm^–2^ for *C. colocynthis* and 29.4±4.2 µg cm^–2^ for *P. dactylifera*. The cuticular waxes of both plant species were mainly composed of VLC aliphatic compounds (Fig. 5). VLC aliphatic compounds with carbon chain-lengths ranging from C_20_ to C_46_ amounted to 90% of total cuticular waxes in *C. colocynthis* (Fig. 6A), and the main constituents were octacosan-1-ol (C_28;_ 13% of total cuticular waxes), triacontan-1-ol (C_30;_ 26%), and dotriacontan-1-ol (C_32_; 13%). In *P. dactylifera,* the VLC aliphatic compounds with carbon chain-lengths ranging from C_20_ to C_62_ amounted to 88% of total cuticular waxes (Fig. 6B), with dotriacontanoic acid (C_32_; 15% of total cuticular waxes) and alkyl esters with carbon chain-length C_48_ (10%), C_56_ (6%), and C_58_ (6%) as the dominant constituents (Supplementary Table S2). Only a minor fraction of cuticular waxes contained cyclic compounds in both plant species (<1% of total cuticular waxes in *C. colocynthis* and 4% in *P. dactylifera*).

### Chemical composition of the cutin matrix

The coverage of the cutin monomers of *C. colocynthis* leaves was 7.8±1.3 µg cm^–2^ and that of *P. dactylifera* leaflets 57.4±4.2 µg cm^–2^. For both plant species, the cutin matrix was composed of ~88% aliphatic and ~12% aromatic cutin monomers. For the leaf cutin of *C. colocynthis*, the carbon chain-lengths of aliphatic compounds ranged from C_16_ to C_32_, but for *P. dactylifera* leaflets they ranged up to C_34_. The ratio of the cutin acids with a carbon chain-length of C_16_ and C_18_ was 6:1 for *C. colocynthis* leaves and 1:11 for *P. dactylifera* leaflets. The predominant cutin acid was 9/10,16-dihydroxy hexadecanoic acid (51% of total cutin monomers) in *C. colocynthis* leaves and 18-hydroxy 9,10-epoxy octadecanoic acid (60%) in *P. dactylifera* leaflets. The 9,10-epoxy 18-hydroxy octadecanoic acid was absent from the *C. colocynthis* leaf cutin. The aromatic cutin constituent was mainly *trans*-4-hydroxycinnamic acid, representing 7% of total cutin monomers in both *C. colocynthis* and *P. dactylifera* (Supplementary Table S3).

### Melting behaviour of cuticular waxes

**Fig. 7.**
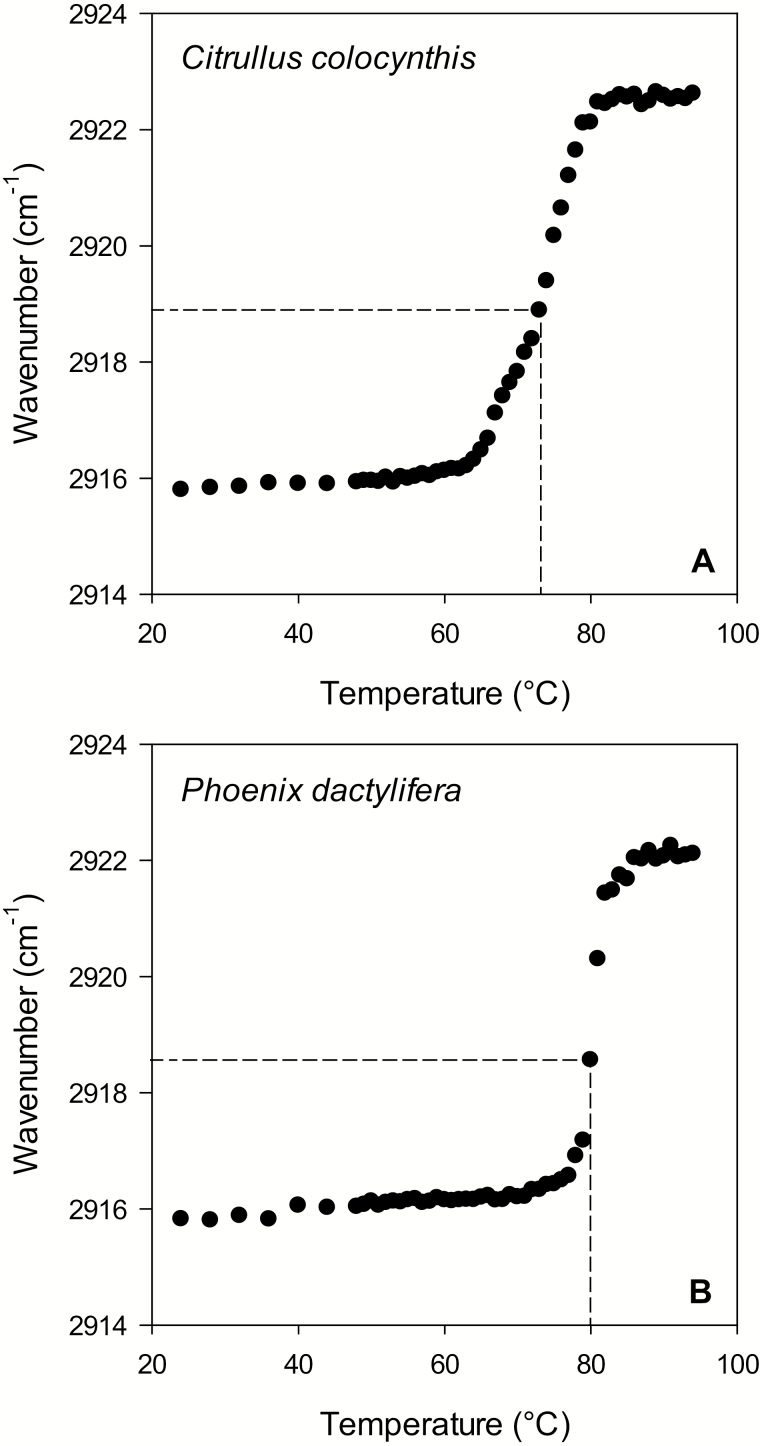
Melting curves of cuticular waxes of *Citrullus colocynthis* leaves (A) and *Phoenix dactylifera* leaflets (B) derived from the temperature-dependent shift of the absorption maxima of the asymmetric CH_2_ stretching modes. Dashed lines represent the midpoints of wax melting, which correspond to the 50% frequency change.

The melting ranges of the leaf cuticular waxes of *C. colocynthis* and *P. dactylifera* were determined by FTIR. The wavenumbers of the maxima of the CH_2_ asymmetric stretching band (ν_(as)_) were plotted against temperature. In long-chain alkyl crystals the wavenumbers of the maxima of ν_(as)_ increase with temperature as a consequence of increasing disorder of the C-C chain (Snyder *et al.*, 1982). This disorder reaches its maximum when the long-chain alkyl sample is entirely liquid. Since waxes are multicomponent materials, they do not display a sharp melting point, but more or less extended melting ranges (Merk *et al.*, 1997). *C. colocynthis* wax melted in the temperature range from 60 °C to 80 °C, while the wax of *P. dactylifera* started to melt at ~70 °C and wholly melted at 88 °C. The midpoints of the wax melting ranges were identified as the temperatures where 50% of the total frequency shift was reached. The midpoints of the melting ranges of *P. dactylifera* and *C. colocynthis* waxes were 80 °C and 73 °C, respectively (Fig. 7).

## Discussion

Water-saver and water-spender plants growing in hot arid environments are characterized by common syndromes of traits on the morphological, physiological, biochemical, cellular, and molecular levels allowing them to cope with the unique challenges of such habitats. The intention of our study of *C. colocynthis* and *P. dactylifera* was to investigate whether the efficacy of the cuticular transpiration barrier against water loss and its susceptibility to thermal damage are as yet unidentified traits pertaining to the water-saver and water-spender strategies.

Morphological leaf traits are very often directly related to life strategies. The LMA reflects the trade-off between carbon gain and longevity (Díaz *et al.*, 2016), while the LWC roughly indicates leaf density (Garnier and Laurent, 1994). The evergreen leaflets of *P. dactylifera* have an LMA three times higher than the leaves of *C. colocynthis*, indicating the much higher investment into the long-lived fronds of *P. dactylifera*. The comparably high costs for producing long-lived evergreen leaves are the price to be paid for a higher survival rate in the face of abiotic and biotic hazards (Coley *et al.*, 1985; Reich *et al.*, 1997; Wright *et al.*, 2004). Hence, the high LMA and low LWC confer higher stress resistance in *P. dactylifera* compared with *C. colocynthis*.

**Fig. 8.**
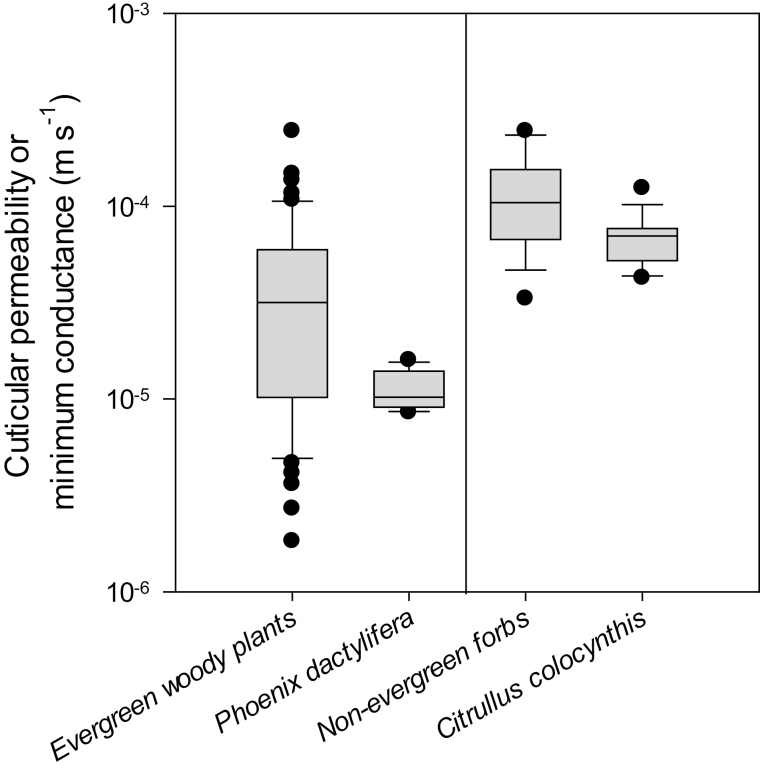
Comparison of the minimum conductances at 25 °C measured in leaves of *P. dactylifera* and *C. colocynthis* with minimum conductances or cuticular permeabilities from the corresponding ecological groups of evergreen woody plants (*n*=54) and non-evergreen forbs (*n*=18). Data for these two latter groups were taken from Table S1 in Schuster *et al.* (2017).

In the leaf drying experiments, the minimum conductance as a proxy for cuticular transpiration of *P. dactylifera* and *C. colocynthis* was measured. For *C. colocynthis*, a *g*_min_ of 6.9±2.0×10^–5^ m s^–1^ at 25 °C was found, which lies at the lower end of the interquartile range of cuticular water permeabilities or minimum conductances measured so far for non-evergreen forbs from temperate climates (Fig. 8). The *g*_min_ of *C. colocynthis* is very close to the *g*_min_ of the slightly xerophytic species *Hippocrepis comosa*, *Sanguisorba minor*, and *Salvia pratensis* from temperate climates (Schuster, 2016). The *g*_min_ of *P. dactylifera* leaflets is about six times lower but is still at the lower end of the interquartile range formerly reported for evergreen woody plants from various climates (Fig. 8). Thus, the cuticles of the two hot desert species studied, while being low, do not provide, at mild temperatures, particularly efficient protection against non-stomatal leaf water loss as might be intuitively expected for plants living in such habitats.

The RWD_SC_ of *C. colocynthis* was 0.2 at 25 °C and drastically increased above 40 °C to ~0.6 at 50 °C, indicating that at high temperatures, leaf desiccation proceeds to a greater degree before the stomata close. This agrees well with field studies showing that the cooling effect of stomatal transpiration keeps the leaf temperatures below the thermal tolerance of this plant species (46 °C), avoiding death by overheating (Lange, 1959). Allowing the leaf water potential to decrease maintains the water flow from the soil to compensate for the water loss by stomatal transpiration that is necessary for leaf cooling. Thus, *C. colocynthis* employs a typical strategy of water-spender plants adapted to heat stress (Lange, 1959; Althawadi and Grace, 1986; Beerling *et al.*, 2001; Schulze *et al.*, 2005). In contrast to this behaviour, Lange (1959) did not find a cooling effect with *P. dactylifera*. The leaflet temperature was up to 11 °C above that of the air, and the transpiration rates were almost unnoticeable with the method used by this author in the field. The RWD_SC_ in *P. dactylifera* is 10 times lower than for *C. colocynthis* and remains low over the whole temperature range measured (Table 2). Hence, *P. dactylifera* is very sensitive to leaf water loss and closes its stomata early in the course of desiccation (Müller *et al.* 2017). *P. dactylifera* closes its stomata at small changes in the RWD at all experienced temperatures, thus preventing leaf damage, and has a low g_min_, minimizing water loss. Both features are typical for water-saver plants, irrespective of their phylogeny (Guehl and Aussenac, 1987; Lo Gullo and Salleo, 1988; Baquedano and Castillo, 2006).

Being a kinetic phenomenon, the diffusion velocity of water in the cuticular transpiration barrier rises with temperature, which in all cases studied so far also led to an increase in cuticular permeability (Schreiber, 2002; Riederer, 2006). The temperature dependence of a kinetic parameter can be analysed quantitatively and mechanistically using the Arrhenius formalism by plotting the natural logarithm of the rate constant—in this case, the cuticular permeability—versus the inverse absolute temperature. An abrupt increase or decrease in the slope of the Arrhenius plot at a specific temperature indicates a change in the properties of the surroundings, on a molecular scale, of the diffusing water molecule. The Arrhenius formalism applied to the cuticular permeance of *C. colocynthis* leaves led to two distinct linear plots for the lower and the higher temperature ranges (Fig. 4B): (i) no significant change of permeance at lower temperatures ranging from 25 °C to 35 °C and (ii) a sharp increase at higher temperatures from 40 °C to 50 °C, with a transition temperature of 37.7 °C. This implies that at higher temperatures the water molecules diffuse through pathways that are not accessible at lower temperatures. The activation energy (*E*_p_) of cuticular permeation of *C. colocynthis* derived from the slope of the Arrhenius plot at higher temperatures (for details of the calculations see Riederer, 2006) is 76.3±33.4 kJ mol^–1^. This activation energy coincides with the range of the respective values reported so far in the literature (Riederer, 2006).

In contrast to *C. colocynthis*, the Arrhenius plot for the minimum conductance of *P. dactylifera* is linear and parallel to the x-axis over the whole range of temperatures studied. Permeability does not change from 25 °C to 50 °C, and no activation energy can be estimated. This pronounced resistance to a thermally induced deterioration of the cuticular transpiration barrier is comparable to that recently described for the hot-desert shrub *R. stricta* (Schuster *et al.*, 2016), suggesting that resistance to substantial thermal effects on cuticular permeability is an adaptive trait of desert water-saver plants from hot arid habitats.

What is the basis for the observed difference in the thermal susceptibility of the cuticular transpiration barrier of the water-saver plant *P. dactylifera* and the water-spender *C. colocynthis*? The differences can be assumed to be related to the chemical composition and physical structure of the cuticle of the two species, especially differences in the qualitative and quantitative composition of the main cuticular components—cutin and cuticular waxes—and the aggregates they form. The cutin matrix of both plant species mainly consisted of ester-cross-linked C_16_ and C_18_ hydroxy alkanoic acids, predominantly 9/10,16-dihydroxy hexadecanoic acids in *C. colocynthis* leaves and 18-hydroxy-9,10-epoxy octadecanoic acid in *P. dactylifera* leaflets. The cutin composition of *C. colocynthis* leaves represents the C_16_ type, and of *P. dactylifera* leaflets the C_18_ type, of cutin, according to the classification proposed by Holloway (1982). The amount of cutin monomers per unit surface area was seven times lower in *C. colocynthis* leaves compared with *P. dactylifera* leaflets. Differences in the amount and composition of cutin have been discussed as the cause of differences in the degree of cross-linking of the polymeric network and, consequently, in the matrix structure and the viscoelastic properties of the cutin (Fich *et al.*, 2016; Khanal and Knoche, 2017). The mid-chain epoxy groups of the 18-hydroxy-9,10-epoxy octadecanoic acid dominating the cutin of *P. dactylifera* can form very stable ether bonds conferring particular robustness on the cuticle of this species (Schmidt and Schönherr, 1982; Riederer and Schönherr, 1988), which in turn may decrease the thermal vulnerability of its cuticular transpiration barrier. Schreiber and Schönherr (1990) hypothesized that discrepancies in the thermoelastic properties of cutin and waxes might cause defects in the cuticular transpiration barrier, leading to a steep increase of its water permeability above a specific transition temperature.

Another reason for the divergence in the thermal susceptibility of the cuticular transpiration barriers of the two species may lie in differences in the cuticular wax composition, specifically in the VLC aliphatic part, which has been shown to be the primary factor determining cuticular water permeability (Vogg *et al.*, 2004; Leide *et al.*, 2007, 2011; Buschhaus and Jetter, 2012; Jetter and Riederer, 2016). A model proposed for the molecular structure of VLC cuticular waxes by Riederer and Schreiber (1995) states that cuticular waxes are multiphase systems composed of highly ordered crystalline lamellae interspersed with amorphous zones. According to this model, the inner parts of the alkyl chains of the VLC aliphatic molecules make up the crystalline domains of the cuticular waxes, while the irregularly protruding chain ends, which do not fit into the crystallites, form the adjacent amorphous zones. The cuticular waxes of both *P. dactylifera* and *C. colocynthis* fit this model. Although *P. dactylifera* and *C. colocynthis* qualitatively present similar aliphatic compound classes, the quantitative contributions of each class to the total cuticular wax load and the carbon chain-lengths of homologous compounds vary considerably. The cuticular waxes of *P. dactylifera* contain higher amounts of all compound classes except for primary alcohols, which occur in equal quantities. The total VLC wax coverage of the leaflets of *P. dactylifera* was seven times higher than that of *C. colocynthis* leaves.

Although the carbon chain-lengths of the VLC aliphatic compounds range from C_20_ to C_46_ in *C. colocynthis* and from C_20_ to C_62_ in *P. dactylifera*, the overall weighted MCL, which are a proxy for the chain-length distributions, differ by only about three carbon atoms (31.8 in *P. dactylifera* and 29.4 in *C. colocynthis*). However, the patterns of the respective chain-length distributions of VLC wax components drastically differ between the two species. The VLC aliphatic compounds of both species can be divided into two groups: (i) components with fewer than 40 carbon atoms and (ii) components with chains of 40 or more carbon atoms. *N*-alkanes, *n*-alkenes, *n*-aldehydes, alkanoic acids, and primary and secondary alcohols make up the fraction with chain-lengths <C_40_ whereas the cuticular wax fraction with chain-length ≥C_40_ consists exclusively of VLC alkyl esters from long-chain fatty acids and primary alcohols. The aliphatic components with chain-lengths <C_40_ and ≥C_40_ comprise 69 mol % and 31 mol %, respectively, of the total VLC aliphatic fraction in *P. dactylifera*. In contrast, only a minor fraction (1 mol %) of the VLC aliphatic components was ≥C_40_ in *C. colocynthis*, while almost all compounds belonged to the shorter chain-length fraction of the cuticular waxes (Fig. 6). The MCL of the shorter-chain components is 29.4 in *C. colocynthis* and 31.3 in *P. dactylifera*, while the MCL of the VLC ester fraction in *P. dactylifera* is 51.3 (Fig. 6).

We hypothesize that the high proportion of VLC aliphatic compounds ≥C_40_ in *P. dactylifera* significantly affects the physical properties of the leaf cuticular transpiration barrier, thereby leading to more efficient control of cuticular water loss over the range of temperatures experienced by this plant in its natural habitat. The reason for this particular thermal stability may be the high melting temperature of cuticular waxes, which consist to a considerable degree of VLC alkyl esters. Indeed, the melting temperatures of cuticular waxes as determined by FTIR differ significantly between the two species, with midpoints of the solid–liquid transition occurring at 73 °C in *C. colocynthis* and 80 °C in *P. dactylifera* (Fig. 7). This finding shows that waxes of both species differ in their susceptibility to thermally induced changes to their physical properties. Based on evidence obtained from the study of technical waxes, we propose that the presence of appreciable amounts of VLC alkyl esters in the cuticular waxes of *P. dactylifera* affects the melting range of the cuticular wax in a particular way. It was shown that VLC aliphatic esters intercalate simultaneously into two adjacent crystalline lamellae formed by the shorter-chain VLC aliphatic compounds of a wax (Reynhardt and Riederer, 1994; Dorset, 1999, 2002, 2005) under the condition that the chain-length of the esters is appreciably higher than that of the shorter chain-length fraction. This condition is fulfilled in the case of *P. dactylifera* wax, with an MCL of 51.3 for the longer-chain fraction and 31.3 for the shorter-chain fraction.

**Fig. 9.**
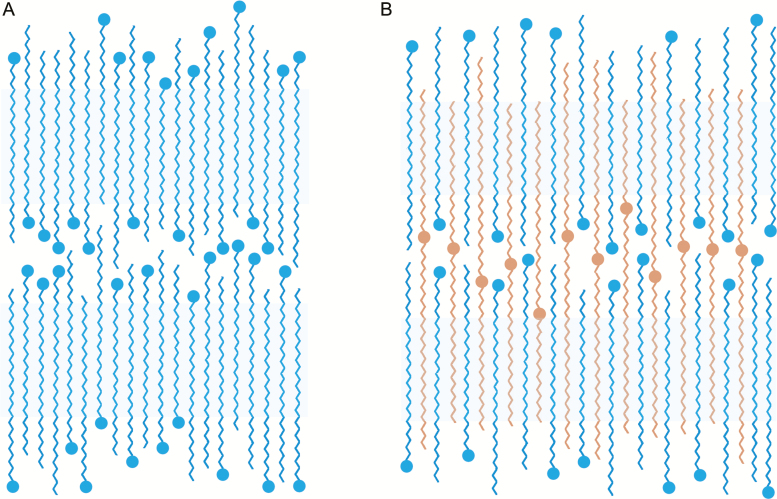
Model proposed for the molecular structure of the cuticular waxes of *Citrullus colocynthis* leaves (A) and *Phoenix dactylifera* leaflets (B). The inner parts of the alkyl chains of very-long-chain (VLC) aliphatic molecules (blue zigzag lines) form the highly ordered crystalline domains (blue background), whereas the chain ends make up the adjacent amorphous zones of the cuticular waxes. Dots symbolize functional groups. The absolute chain-lengths and their distribution represent the corresponding experimental values for both species, with weighted median chain-lengths of 29.4 for *C. colocynthis* and 31.8 for *P. dactylifera*. The VLC alkyl esters (orange zigzag lines) integrate within the two crystalline lamellae. These bridging molecules form covalent anchors connecting two adjacent crystalline flakes.

Consequently, a representative ester molecule of 51 C atoms will be integrated simultaneously by up to 25 C atoms each into the two adjacent lamellae composed of molecules with an MCL of 31. These bridging molecules represent covalent anchors holding the crystalline lamellae together (Fig. 9B) and thereby increase the melting point of the cuticular waxes. The tight bridging of the crystalline lamellae also affects the extent of the effect temperature has on the mechanical properties of the wax (Reynhardt and Riederer, 1994). The thermal expansion coefficient of the leaf cuticular wax of the palm *Copernicia prunifera* (Mill.) H.E. Moore (carnauba wax), which has an overall composition and an alkyl ester content equal to that of *P. dactylifera* (Basson and Reynhardt, 1988), is much lower than the coefficient for waxes without VLC alkyl esters (Craig *et al.*, 1965).

Further, paraffin waxes can be mechanically reinforced by the addition of VLC esters. To quantify this effect, the mechanical deformability of paraffin with varying amounts of carnauba wax admixed was measured as a function of temperature by Craig *et al*. (1966). The addition of 25% of carnauba wax, which leads to a VLC ester content of only 8% in the final mixture, increased the temperature at which the mixed wax could be deformed by a given load by ~10 °C (Craig *et al.*, 1966).

We hypothesize that the absence of an effect of temperature on the cuticular water permeability of *P. dactylifera*, in contrast to that seen with *C. colocynthis*, is due to a higher and less temperature-sensitive mechanical stability of the alkyl ester-containing waxes of the former species, even under elevated temperatures. The presence of alkyl ester-containing ‘bridged’ waxes can be imagined to mechanically reinforce the cuticular transpiration barrier of *P. dactylifera* and, thus, prevent the occurrence of thermally induced structural defects that are thought to lead to the abrupt increases of water permeability at higher temperatures observed in *C. colocynthis* and many other species (Schreiber, 2001; Riederer, 2006). We further hypothesize that the production of alkyl ester-reinforced cuticular waxes is particularly beneficial to plant species living in hot environments, and that this is comparable to the advantage the desert plant *R. stricta* is assumed to derive from the incorporation of appreciable amounts of pentacyclic triterpenoids into its cutin matrix (Schuster *et al.*, 2016). The hypotheses we propose here are testable. Therefore, investigations into the phase behaviour and the effect of temperature on the mechanical and rheological properties of cuticular waxes of varying compositions and chain-length distributions are ongoing.

In conclusion, our findings show for the first time that a plant exhibiting the water-saver strategy such as *P. dactylifera* differs from a water-spender plant like *C. colocynthis* not only in terms of the response of stomatal regulation to water shortage but also by a constitutive difference in cuticular barrier properties. The cuticle of the water-saver *P. dactylifera* (i) more efficiently reduces cuticular transpirational water loss and (ii) does not experience thermal damage even under the impact of high temperatures up to 50 °C. The cuticular waxes play an essential role in the efficacy and stability of the transpirational barrier to water loss. We hypothesize that the cuticular transpiration barrier of *P. dactylifera* leaves is thermally resistant because an appreciable fraction of the wax consists of VLC alkyl esters, which bridge the crystalline lamellae of the cuticular waxes and, thus, reinforce the transport-limiting barrier.

## Supplementary data

Supplementary data are available at *JXB* online.

Table S1. Minimum transpiration (*J*_min_) and leaf to air temperature difference (Δ*T*_leaf–air_) of *Citrullus colocynthis* leaves and *Phoenix dactylifera* leaflets obtained from drying curves as functions of air temperatures (*T*_air_).

Table S2. Cuticular wax coverage and components of *Citrullus colocynthis* leaves and *Phoenix dactylifera* leaflets.

Table S3. Cutin monomeric coverage of *Citrullus colocynthis* leaves and *Phoenix dactylifera* leaflets.

Figure S1. Representative transpiration kinetic curves of *Citrullus colocynthis* leaves and *Phoenix dactylifera* leaflets.

Supplementary Table S1-S3

Supplementary Figure S1

## Notes

In memory of Otto Ludwig Lange (1927–2017).

## Abbreviations:

FTIR: Fourier transform infrared spectroscopy
*g*_min_: minimum leaf conductance
MCL: weighted median carbon chain-lengths
RWD: relative water deficit
RWD_SC_: relative water deficit at maximum stomatal closure
VLC: very-long-chain

## References

[CIT0001] Almazroui M, Islam MN, Dambul R, Jones PD 2014 Trends of temperature extremes in Saudi Arabia. International Journal of Climatology 34, 808–826.

[CIT0002] Althawadi AM, Grace J 1986 Water use by the desert cucurbit *Citrullus colocynthis* (L.) Schrad. Oecologia 70, 475–480. 28311938 10.1007/BF00379514

[CIT0003] Baquedano FJ, Castillo FJ 2006 Comparative ecophysiological effects of drought on seedlings of the Mediterranean water-saver *Pinus halepensis* and water-spenders *Quercus coccifera* and *Quercus ilex*. Trees 20, 689–700.

[CIT0004] Barrow SC 1998 A monograph of *Phoenix* L. (Palmae: Coryphoideae). Kew Bulletin 53, 513–575.

[CIT0005] Basson I, Reynhardt EC 1988 An investigation of the structures and molecular dynamics of natural waxes: II. Carnauba wax. Journal of Physics D: Applied Physics 21, 1429–1433.

[CIT0006] Beerling DJ, Osborne CP, Chaloner WG 2001 Do drought-hardened plants suffer from fever? Trends in Plant Science 6, 507–508. 10.1016/s1360-1385(01)02089-1 11701375

[CIT0007] Burghardt M, Burghardt A, Gall J, Rosenberger C, Riederer M 2008 Ecophysiological adaptations of water relations of *Teucrium chamaedrys* L. to the hot and dry climate of xeric limestone sites in Franconia (Southern Germany). Flora 203, 3–13.

[CIT0008] Burghardt M, Riederer M 2003 Ecophysiological relevance of cuticular transpiration of deciduous and evergreen plants in relation to stomatal closure and leaf water potential. Journal of Experimental Botany 54, 1941–1949. 12815029 10.1093/jxb/erg195

[CIT0009] Burghardt M, Riederer M 2006 Cuticular transpiration. In: Riederer M, Müller C, eds. Biology of the plant cuticle: annual plant reviews. Oxford: Blackwell Publishing, 192–311.

[CIT0010] Buschhaus C, Jetter R 2012 Composition and physiological function of the wax layers coating Arabidopsis leaves: β-amyrin negatively affects the intracuticular water barrier. Plant Physiology 160, 1120–1129. 22885935 10.1104/pp.112.198473 PMC3461534

[CIT0011] Coley PD, Bryant JP, Chapin FS 3rd 1985 Resource availability and plant antiherbivore defense. Science 230, 895–899. 17739203 10.1126/science.230.4728.895

[CIT0012] Craig RG, Eick JD, Peyton FA 1965 Properties of natural waxes used in dentistry. Journal of Dental Research 44, 1308–1316.

[CIT0013] Craig RG, Eick JD, Peyton FA 1966 Flow of binary and tertiary mixtures of waxes. Journal of Dental Research 45, 397–403. 5220628 10.1177/00220345660450023101

[CIT0014] Cunningham GM, Mulham WE, Milthorpe PL, Leigh JH 2011 Plants of western New South Wales. Collingwood: CSIRO Publishing.

[CIT0015] Díaz S, Kattge J, Cornelissen JH, et al 2016 The global spectrum of plant form and function. Nature 529, 167–171. 26700811 10.1038/nature16489

[CIT0016] Dorset DL 1999 Bridged lamellae: crystal structure(s) of low molecular weight linear polyethylene. Macromolecules 32, 162–166.

[CIT0017] Dorset DL 2002 From waxes to polymers—crystallography of polydisperse chain assemblies. Structural Chemistry 13, 329–337.

[CIT0018] Dorset DL 2005 Crystallography of the polymethylene chain: an inquiry into the structure of waxes. Oxford: Oxford University Press.

[CIT0019] Fich EA, Segerson NA, Rose JK 2016 The plant polyester cutin: biosynthesis, structure, and biological roles. Annual Review of Plant Biology 67, 207–233. 10.1146/annurev-arplant-043015-111929 26865339

[CIT0020] Garnier E, Laurent G 1994 Leaf anatomy, specific mass and water content in congeneric annual and perennial grass species. New Phytologist 128, 725–736.

[CIT0021] Guehl JM, Aussenac G 1987 Photosynthesis decrease and stomatal control of gas exchange in *Abies alba* Mill. in response to vapor pressure difference. Plant Physiology 83, 316–322. 16665243 10.1104/pp.83.2.316 PMC1056355

[CIT0022] Holloway PJ 1982 The chemical constitution of plant cutins. In: Cutler DF, Alvin KL, Price CE, eds. The plant cuticle. London: Academic Press, 45–85.

[CIT0023] Jetter R, Kunst L, Samuels AL 2006 Composition of plant cuticular waxes. In: Riederer M, Müller C, eds. Biology of the plant cuticle: annual plant reviews. Oxford: Blackwell Publishing, 145–181.

[CIT0024] Jetter R, Riederer M 2016 Localization of the transpiration barrier in the epi- and intracuticular waxes of eight plant species: water transport resistances are associated with fatty acyl rather than alicyclic components. Plant Physiology 170, 921–934. 26644508 10.1104/pp.15.01699 PMC4734581

[CIT0025] Khanal BP, Knoche M 2017 Mechanical properties of cuticles and their primary determinants. Journal of Experimental Botany 68, 5351–5367. 28992090 10.1093/jxb/erx265

[CIT0026] Körner C 1995 Leaf diffusive conductances in the major vegetation types of the globe. In: Schulze ED, Caldwell MM, eds. Ecophysiology of photosynthesis. Berlin: Springer, 463–490.

[CIT0027] Lange OL 1959 Untersuchungen über Wärmehaushalt und Hitzeresistenz mauretanischer Wüsten-und Savannenpflanzen. Flora 147, 595–651.

[CIT0028] Leide J, Hildebrandt U, Reussing K, Riederer M, Vogg G 2007 The developmental pattern of tomato fruit wax accumulation and its impact on cuticular transpiration barrier properties: effects of a deficiency in a beta-ketoacyl-coenzyme A synthase (LeCER6). Plant Physiology 144, 1667–1679. 17468214 10.1104/pp.107.099481 PMC1914139

[CIT0029] Leide J, Hildebrandt U, Vogg G, Riederer M 2011 The *positional sterile* (*ps*) mutation affects cuticular transpiration and wax biosynthesis of tomato fruits. Journal of Plant Physiology 168, 871–877. 21242016 10.1016/j.jplph.2010.11.014

[CIT0030] Lo Gullo MA, Salleo S 1988 Different strategies of drought resistance in three *Mediterranean sclerophyllous* trees growing in the same environmental conditions. New Phytologist 108, 267–276. 10.1111/j.1469-8137.1988.tb04162.x 33873932

[CIT0031] Merk S, Blume A, Riederer M 1997 Phase behaviour and crystallinity of plant cuticular waxes studied by Fourier transform infrared spectroscopy. Planta 204, 44–53.

[CIT0032] Müller HM, Schäfer N, Bauer H, et al 2017 The desert plant *Phoenix dactylifera* closes stomata via nitrate-regulated SLAC1 anion channel. New Phytologist 216, 150–162. 28670699 10.1111/nph.14672

[CIT0033] Nobel PS 2009 Physicochemical and environmental plant physiology. Oxford: Academic Press.

[CIT0034] Reich PB, Walters MB, Ellsworth DS 1997 From tropics to tundra: global convergence in plant functioning. Proceedings of the National Academy of Sciences, USA 94, 13730–13734. 10.1073/pnas.94.25.13730 PMC28374 9391094

[CIT0035] Reynhardt EC, Riederer M 1994 Structure and molecular dynamics of plant waxes. European Biophysics Journal 23, 59–70.

[CIT0036] Riederer M 2006 Thermodynamics of the water permeability of plant cuticles: characterization of the polar pathway. Journal of Experimental Botany 57, 2937–2942. 16873453 10.1093/jxb/erl053

[CIT0037] Riederer M, Schreiber L 1995 Waxes – the transport barriers of plant cuticles. In: Hamilton RJ, ed. Waxes: chemistry, molecular biology and functions. Dundee: The Oily Press, 131–156.

[CIT0038] Riederer M, Schreiber L 2001 Protecting against water loss: analysis of the barrier properties of plant cuticles. Journal of Experimental Botany 52, 2023–2032. 11559738 10.1093/jexbot/52.363.2023

[CIT0039] Riederer M, Schönherr J 1988 Development of plant cuticles: fine structure and cutin composition of *Clivia miniata* Reg. leaves. Planta 174, 127–138. 24221429 10.1007/BF00394885

[CIT0040] Schmidt HW, Schönherr J 1982 Development of plant cuticles: occurrence and role of non-ester bonds in cutin of *Clivia miniata* Reg. leaves. Planta 156, 380–384. 24272585 10.1007/BF00397478

[CIT0041] Schönherr J 1976 Water permeability of isolated cuticular membranes: The effect of cuticular waxes on diffusion of water. Planta 131, 159–164. 24424766 10.1007/BF00389989

[CIT0042] Schönherr J, Lendzian K 1981 A simple and inexpensive method of measuring water permeability of isolated plant cuticular membranes. Zeitschrift für Pflanzenphysiologie 102, 321–327.

[CIT0043] Schreiber L 2001 Effect of temperature on cuticular transpiration of isolated cuticular membranes and leaf discs. Journal of Experimental Botany 52, 1893–1900. 11520878 10.1093/jexbot/52.362.1893

[CIT0044] Schreiber L 2002 Co-permeability of ^3^H‐labelled water and ^14^C‐labelled organic acids across isolated *Prunus laurocerasus* cuticles: effect of temperature on cuticular paths of diffusion. Plant Cell and Environment 25, 1087–1094.

[CIT0045] Schreiber L, Riederer M 1996 Ecophysiology of cuticular transpiration: comparative investigation of cuticular water permeability of plant species from different habitats. Oecologia 107, 426–432. 28307383 10.1007/BF00333931

[CIT0046] Schreiber L, Schönherr J 1990 Phase transitions and thermal expansion coefficients of plant cuticles: The effects of temperature on structure and function. Planta 182, 186–193. 24197094 10.1007/BF00197109

[CIT0047] Schulze ED, Beck E, Müller-Hohenstein K 2005 Plant ecology. Berlin: Springer.

[CIT0048] Schuster A-C 2016 Chemical and functional analyses of the plant cuticle as leaf transpirational barrier. PhD Thesis Würzburg: Würzburg University.

[CIT0049] Schuster A-C, Burghardt M, Alfarhan A, Bueno A, Hedrich R, Leide J, Thomas J, Riederer M 2016 Effectiveness of cuticular transpiration barriers in a desert plant at controlling water loss at high temperatures. AoB Plants 8, plw027. 27154622 10.1093/aobpla/plw027 PMC4925923

[CIT0050] Schuster AC, Burghardt M, Riederer M 2017 The ecophysiology of leaf cuticular transpiration: are cuticular water permeabilities adapted to ecological conditions? Journal of Experimental Botany 68, 5271–5279. 29036342 10.1093/jxb/erx321

[CIT0051] Si Y, Zhang C, Meng S, Dane F 2009 Gene expression changes in response to drought stress in *Citrullus colocynthis*. Plant Cell Reports 28, 997–1009. 19415285 10.1007/s00299-009-0703-5

[CIT0052] Slavík B 1974 Methods of studying plant water relations. Berlin: Springer.

[CIT0053] Smith SD, Monson RK, Anderson JE 1997 Physiological ecology of North American desert plants. Berlin: Springer.

[CIT0054] Snyder RG, Strauss HL, Elliger CA 1982 Carbon-hydrogen stretching modes and the structure of *n*-alkyl chains. 1. Long, disordered chains. The Journal of Physical Chemistry 86, 5145–5150.

[CIT0055] Vogg G, Fischer S, Leide J, Emmanuel E, Jetter R, Levy AA, Riederer M 2004 Tomato fruit cuticular waxes and their effects on transpiration barrier properties: functional characterization of a mutant deficient in a very-long-chain fatty acid beta-ketoacyl-CoA synthase. Journal of Experimental Botany 55, 1401–1410. 15133057 10.1093/jxb/erh149

[CIT0056] Wright IJ, Reich PB, Westoby M, et al 2004 The worldwide leaf economics spectrum. Nature 428, 821–827. 15103368 10.1038/nature02403

