# Supplementary material for: Effects of temperature on the cuticular transpiration barrier of two desert plants with water-spender and water-saver strategies
Source: J Exp Bot. 2019 Jan 30;70(5):1613–25. doi: 10.1093/jxb/erz018 (PMC6416792; doi:10.1093/jxb/erz018)
Supplement: Supplementary Table S1-S3 [file erz018_suppl_supplementary_table_s1-s3.pdf]

Supplementary Table S1. Minimum transpiration (J_{min}) and leaf to air temperature difference ($\Delta T_{leaf\text{-}air}$) of *Citrullus colocynthis* leaves and *Phoenix dactylifera* leaflets obtained from drying curves as functions of air temperatures (T_{air}). Each value represents the mean ± standard deviation ($n \geq 8$).

T_{air} (°C)	$J_{min} \times 10^3$ (g m^{-2} s^{-1})		$\Delta T_{leaf\text{-}air}$ (°C)	
	C. colocynthis	*P. dactylifera*	*C. colocynthis*	*P. dactylifera*
25	1.27 ± 0.37	0.21 ± 0.04	-0.47 ± 0.15	-0.33 ± 0.13
30	1.85 ± 0.29	0.25 ± 0.04	-0.50 ± 0.15	-0.56 ± 0.11
35	2.54 ± 0.54	0.40 ± 0.05	-0.88 ± 0.20	-1.04 ± 0.33
40	3.83 ± 0.99	0.45 ± 0.12	-1.02 ± 0.54	-1.30 ± 0.69
45	6.87 ± 2.38	0.65 ± 0.13	-2.34 ± 0.61	-1.83 ± 1.40
50	13.16 ± 2.80	1.04 ± 0.21	-3.64 ± 0.45	-1.47 ± 0.10

Supplementary Table S2. The cuticular wax coverage and components of *Citrullus colocynthis* leaves and *Phoenix dactylifera* leaflets. Each value represents the mean ± standard deviation ($n \geq 3$).

Compound class	Carbon chain-length	Wax coverage (µg cm^{-2})	
		C. colocynthis	*P. dactylifera*
alkanoic acids	20	0.003 ± 0.001	-
	21	0.001 ± 0.001	0.005 ± 0.001
	22	0.014 ± 0.020	0.023 ± 0.003
	23	-	0.012 ± 0.003
	24	0.014 ± 0.002	0.088 ± 0.043
	25	0.005 ± 0.003	0.019 ± 0.008
	26	0.022 ± 0.004	0.104 ± 0.025
	27	0.009 ± 0.004	0.041 ± 0.020
	28	0.055 ± 0.016	0.273 ± 0.054
	29	0.019 ± 0.004	0.096 ± 0.025
	30	0.176 ± 0.073	0.581 ± 0.134
	31	0.019 ± 0.007	0.357 ± 0.069
	32	0.128 ± 0.068	4.519 ± 1.735
	33	0.006 ± 0.001	0.173 ± 0.043
	34	0.018 ± 0.010	1.264 ± 0.230
	36	0.010 ± 0.001	-
primary alcohols	22	0.002 ± 0.001	0.002 ± 0.000
	24	0.007 ± 0.002	0.014 ± 0.003
	25	0.002 ± 0.001	0.007 ± 0.003
	26	0.047 ± 0.012	0.072 ± 0.015
	27	0.018 ± 0.003	0.014 ± 0.005
	28	0.563 ± 0.063	0.260 ± 0.114
	29	0.081 ± 0.007	0.013 ± 0.010
	30	1.101 ± 0.122	0.162 ± 0.035
	31	0.055 ± 0.005	0.169 ± 0.022
	32	0.543 ± 0.048	1.223 ± 0.401
	33	0.024 ± 0.002	0.103 ± 0.007
	34	0.123 ± 0.014	0.574 ± 0.188
	35	0.007 ± 0.001	-
	36	0.046 ± 0.007	-
alkyl esters	40	-	0.029 ± 0.008
	42	0.012 ± 0.002	0.080 ± 0.010
	43	-	0.016 ± 0.006
	44	0.010 ± 0.002	0.116 ± 0.033
	45	-	0.028 ± 0.008

Compound class	Carbon chain-length	Wax coverage ($\mu g\ cm^{-2}$)	
		C. colocynthis	*P. dactylifera*
	46	0.022 ± 0.004	0.376 ± 0.155
	47	-	0.075 ± 0.017
	48	-	2.906 ± 0.262
	49	-	0.124 ± 0.012
	50	-	1.414 ± 0.394
	51	-	0.060 ± 0.022
	52	-	0.440 ± 0.083
	53	-	0.083 ± 0.022
	54	-	0.769 ± 0.028
	55	-	0.128 ± 0.040
	56	-	1.742 ± 0.346
	57	-	0.173 ± 0.052
	58	-	1.616 ± 0.510
	59	-	0.096 ± 0.052
	60	-	0.870 ± 0.323
	61	-	0.041 ± 0.032
	62	-	0.192 ± 0.075
aldehydes	30	0.019 ± 0.003	-
	31	-	0.052 ± 0.031
	32	-	1.111 ± 0.861
	34	-	0.184 ± 0.080
alkenes	26	0.008 ± 0.002	0.020 ± 0.005
	27	-	0.023 ± 0.005
	28	0.092 ± 0.015	0.050 ± 0.018
	29	-	0.094 ± 0.018
	30	0.088 ± 0.010	0.266 ± 0.069
	32	0.047 ± 0.004	0.126 ± 0.051
	34	0.024 ± 0.002	-
n-alkanes	25	0.011 ± 0.006	-
	26	-	0.011 ± 0.002
	27	0.036 ± 0.021	0.023 ± 0.007
	28	0.007 ± 0.002	0.031 ± 0.010
	29	0.095 ± 0.021	0.063 ± 0.031
	30	0.009 ± 0.001	0.103 ± 0.030
	31	0.110 ± 0.012	1.118 ± 0.308
	32	-	0.074 ± 0.031
	33	0.040 ± 0.004	0.557 ± 0.047
secondary alcohols	27	0.003 ± 0.001	-
	31	0.014 ± 0.009	0.152 ± 0.047
	33	0.009 ± 0.006	0.065 ± 0.024
total very-long-chain aliphatics		*3.771 ± 0.392*	*25.664 ± 3.654*

Compound class	Carbon chain-length	Wax coverage (µg cm^{-2})	
		C. colocynthis	*P. dactylifera*
alpha-amyrin			0.439 ± 0.322
beta-amyrin		-	0.116 ± 0.092
lupeol		-	0.253 ± 0.054
lupenon		-	0.084 ± 0.060
triterpenoid I		-	0.181 ± 0.048
triterpenoid II		-	0.071 ± 0.041
cholesterol		0.002 ± 0.001	-
total cyclic aliphatics		*0.002 ± 0.001*	*1.145 ± 0.624*
beta-tocopherol		0.005 ± 0.003	-
total cyclic aromatics		*0.005 ± 0.003*	-
total cyclics		0.008 ± 0.003	1.145 ± 0.624
not identified		0.415 ± 0.081	2.530 ± 0.226
total wax		*4.193 ± 0.436*	*29.338 ± 4.235*

Supplementary Table S3. The cutin monomeric coverage of *Citrullus colocynthis* leaves and *Phoenix dactylifera* leaflets. Each value represents the mean value ± standard deviation ($n \geq 5$).

Compound	Carbon chain-length	Cutin coverage (µg cm^{-2})	
		C. colocynthis	*P. dactylifera*
alkanoic acid	16	0.40 ± 0.19	0.55 ± 0.05
alkanoic acid	17	0.03 ± 0.01	-
alkatrienoic acid (9,12,15)	18:3	0.16 ± 0.02	-
alkadienoic acid (9,12)	18:2	0.10 ± 0.02	-
alkenoic acid (9)	18:1	0.09 ± 0.02	-
alkanoic acid	18	0.22 ± 0.06	0.29 ± 0.15
alkanoic acid	20	0.03 ± 0.01	0.01 ± 0.00
alkanoic acid	22	0.05 ± 0.01	0.07 ± 0.01
alkanoic acid	24	0.04 ± 0.01	0.60 ± 0.05
alkanoic acid	26	-	0.61 ± 0.03
alkanoic acid	28	-	0.22 ± 0.02
alkanoic acid	30	0.03 ± 0.01	0.40 ± 0.05
alkanoic acid	32	0.03 ± 0.01	0.12 ± 0.02
alkanoic acid	34	-	0.12 ± 0.03
primary alcohol	16	0.04 ± 0.02	0.07 ± 0.03
primary alcohol	18	0.05 ± 0.01	0.02 ± 0.01
primary alcohol	20	0.06 ± 0.01	0.03 ± 0.01
primary alcohol	22	0.06 ± 0.00	-
primary alcohol	24	0.03 ± 0.01	-
primary alcohol	26	0.01 ± 0.01	0.03 ± 0.01
primary alcohol	28	0.06 ± 0.02	0.03 ± 0.01
primary alcohol	30	0.05 ± 0.01	0.13 ± 0.04
primary alcohol	32	-	0.26 ± 0.02
primary alcohol	34	-	0.03 ± 0.00
9/10-hydroxy alkane-1,16-dioic acid	16	0.40 ± 0.12	-
16-hydroxy alkenoic acid (9)	16:1	0.04 ± 0.01	-
16-hydroxy alkanoic acid	16	0.28 ± 0.03	0.08 ± 0.01
18-hydroxy alkadienoic acid (12,15)	18:2	-	0.66 ± 0.04
18-hydroxy alkadienoic acid (9,12)	18:2	-	1.14 ± 0.08
18-hydroxy alkenoic acid (9)	18:1	-	0.70 ± 0.07
18-hydroxy alkanoic acid	18	0.06 ± 0.01	-
9/10,16-dihydroxy alkanoic acid	16	3.97 ± 0.82	3.11 ± 0.23
9/10,18-dihydroxy alkanoic acid	18	0.23 ± 0.05	0.43 ± 0.05
16-hydroxy 9/10-oxo alkanoic acid	16	-	0.03 ± 0.00
18-hydroxy 9/10-oxo alkanoic acid	18	-	0.25 ± 0.02
18-hydroxy 9,10-epoxy alkanoic acid	18	-	34.28 ± 2.57
9,10,18-trihydroxy alkanoic acid	18	-	4.87 ± 0.92
2-hydroxy alkanoic acid	22	0.06 ± 0.00	0.19 ± 0.02

Compound	Carbon chain-length	Cutin coverage (µg cm^{-2})					
		C. colocynthis			*P. dactylifera*		
2-hydroxy alkanoic acid	23	0.03	±	0.01	0.03	±	0.01
2-hydroxy alkanoic acid	24	0.22	±	0.02	0.31	±	0.10
2-hydroxy alkanoic acid	25	0.04	±	0.01	0.06	±	0.00
2-hydroxy alkanoic acid	26	0.07	±	0.01		-	
cis-4-hydroxy cinnamic acid		0.04	±	0.01	0.39	±	0.07
trans-4-hydroxy cinnamic acid		0.52	±	0.09	3.85	±	0.10
3,4-dihydroxy cinnamic acid		0.22	±	0.11	0.06	±	0.06
4-hydroxy 3-methoxy cinnamic acid		0.01	±	0.00	0.01	±	
3,5-dimethoxy 4-hydroxy cinnamic acid			-		0.28	±	0.09
4-hydroxy benzoic acid			-		1.55	±	0.07
3,4-dihydroxy benzoic acid			-		0.57	±	0.04
4-hydroxy 3-methoxy benzoic acid		0.05	±	0.02	0.18	±	0.02
3-methoxy mandelic acid			-		0.41	±	0.02
4-hydroxy 3-methoxy mandelic acid			-		0.45	±	0.18
total cutin		*7.75*	±	*1.28*	*57.44*	±	*4.18*
