# Supplementary material for: Effects of temperature on the cuticular transpiration barrier of two desert plants with water-spender and water-saver strategies
Source: J Exp Bot. 2019 Jan 30;70(5):1613–25. doi: 10.1093/jxb/erz018 (PMC6416792; doi:10.1093/jxb/erz018)

Supplementary Figure S1. Kinetic curves of water loss from detached leaves of *C. colocynthis* and leaflets of *P. dactylifera* exposed to dry air over time.

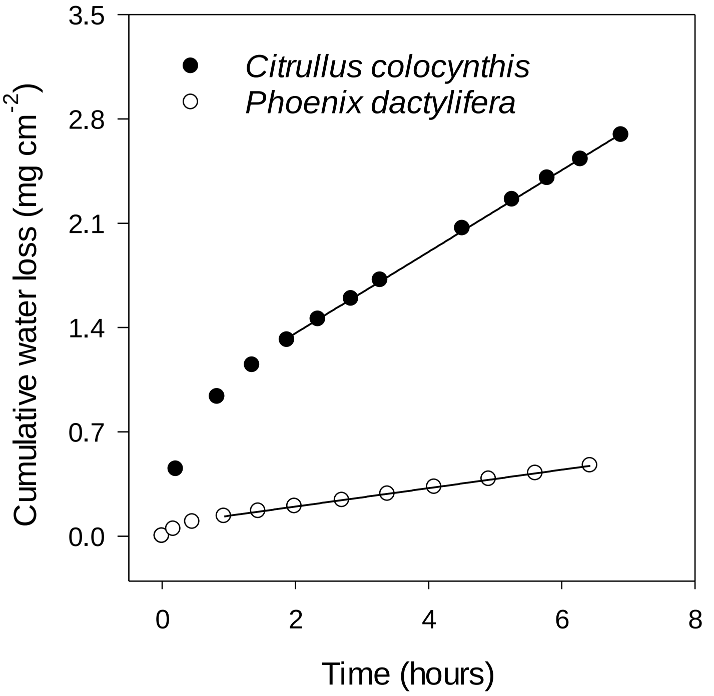

Supplement: Supplementary Figure S1 [file erz018_suppl_supplementary_figure_s1.pdf]
